# Global critical care: a call to action

**DOI:** 10.1186/s13054-022-04296-3

**Published:** 2023-01-20

**Authors:** Ana Maria Crawford, Ananya Abate Shiferaw, Papytcho Ntambwe, Alexei Ortiz Milan, Karima Khalid, Rodrigo Rubio, Francoise Nizeyimana, Fredy Ariza, Alhassan Datti Mohammed, Tim Baker, Paulin Ruhato Banguti, Farai Madzimbamuto

**Affiliations:** 1 Anesthesiology and Critical Care, Stanford University, 300 Pasteur Drive, Stanford, CA USA; 2 College of Health Sciences, Addis Ababa University, Addis Ababa, Ethiopia; 3 Anaesthesia and Critical Care, Livingstone University Teaching Hospital, Livingstone, Zambia; 4 Critical Care Medicine Physician, Sir Ketumile Masire Teaching Hospital, Notwane and Mabutho Road, Plot 4775, Private Bag UB 001, Gaborone, Botswana; 5 Muhimbili University of Health and Allied Sciences, Dar es Salaam, Tanzania; 6 Departamento de Anestesia, Hospital ABC, Vasco de Quiroga 154, Cuajimalpa, 05348 Ciudad de Mexico, Mexico; 7 Consultant Anesthesiology and Critical Care, Head of Department CHUK, Kigali, Rwanda; 8 Anesthesia and Perioperative Medicine, Fundación Valle del Lili, ICESI/UNIVALLE Universities, Cali, Colombia; 9 Department of Anaesthesiology and Intensive Care, Bayero University, Aminu Kano Teaching Hospital, Kano, Nigeria; 10 Department of Global Public Health, Karolinska Institutet, Stockholm, Sweden; 11 Queen Mary University of London, London, UK; 12 Anesthesiology and Critical Care, University of Rwanda, Kigali, Rwanda; 13 Anaesthesiology and Critical Care, University of Botswana School of Medicine, Notwane and Mabutho Road, Plot 4775, Private Bag UB 001, Gaborone, Botswana

**Keywords:** Critical care, Global health, Critical illness, Burden of illness, ICU

## Abstract

Critical care is underprioritized. A global call to action is needed to increase equitable access to care and the quality of care provided to critically ill patients. Current challenges to effective critical care in resource-constrained settings are many. Estimates of the burden of critical illness are extrapolated from common etiologies, but the true burden remains ill-defined. Measuring the burden of critical illness is epidemiologically challenging but is thought to be increasing. Resources, infrastructure, and training are inadequate. Millions die unnecessarily due to critical illness. Solutions start with the implementation of first-step, patient care fundamentals known as Essential Emergency and Critical Care. Such essential care stands to decrease critical-illness mortality, augment pandemic preparedness, decrease postoperative mortality, and decrease the need for advanced level care. The entire healthcare workforce must be trained in these fundamentals. Additionally, physician and nurse specialists trained in critical care are needed and must be retained as leaders of critical care initiatives, researchers, and teachers. Context-specific research is mandatory to ensure care is appropriate for the patient populations served, not just duplicated from high-resourced settings. Governments must increase healthcare spending and invest in capacity to treat critically ill patients. Advocacy at all levels is needed to achieve universal health coverage for critically ill patients.

## A call to action for global critical care

Excellence in critical care occurs when all patients around the globe have access to the care that saves lives and prevents disability. We are far from that reality. In 2015 the Lancet Commission for Global Surgery convened because surgery had yet to be considered an indivisible, indispensable part of Global Health. This commission launched surgery up the global priority list. The world now agrees that surgery is a core component of universal health coverage (UHC), is cost-effective, and is life-preserving. A similar process for critical care is needed.

In less than 3 years, over 6 million people have died due to COVID-19. Yet, even after a pandemic, critical care remains underprioritized and solutions siloed. Vaccines, oxygen, ventilators, diagnostics, and therapeutics are essential to addressing the global burden of critical illness, but all independently fail when infrastructure, systems, provider training, and governmental accountability are lacking. Similar to Global Surgery, UHC cannot be achieved without the care critically ill patients need.

**Fig. 1:**
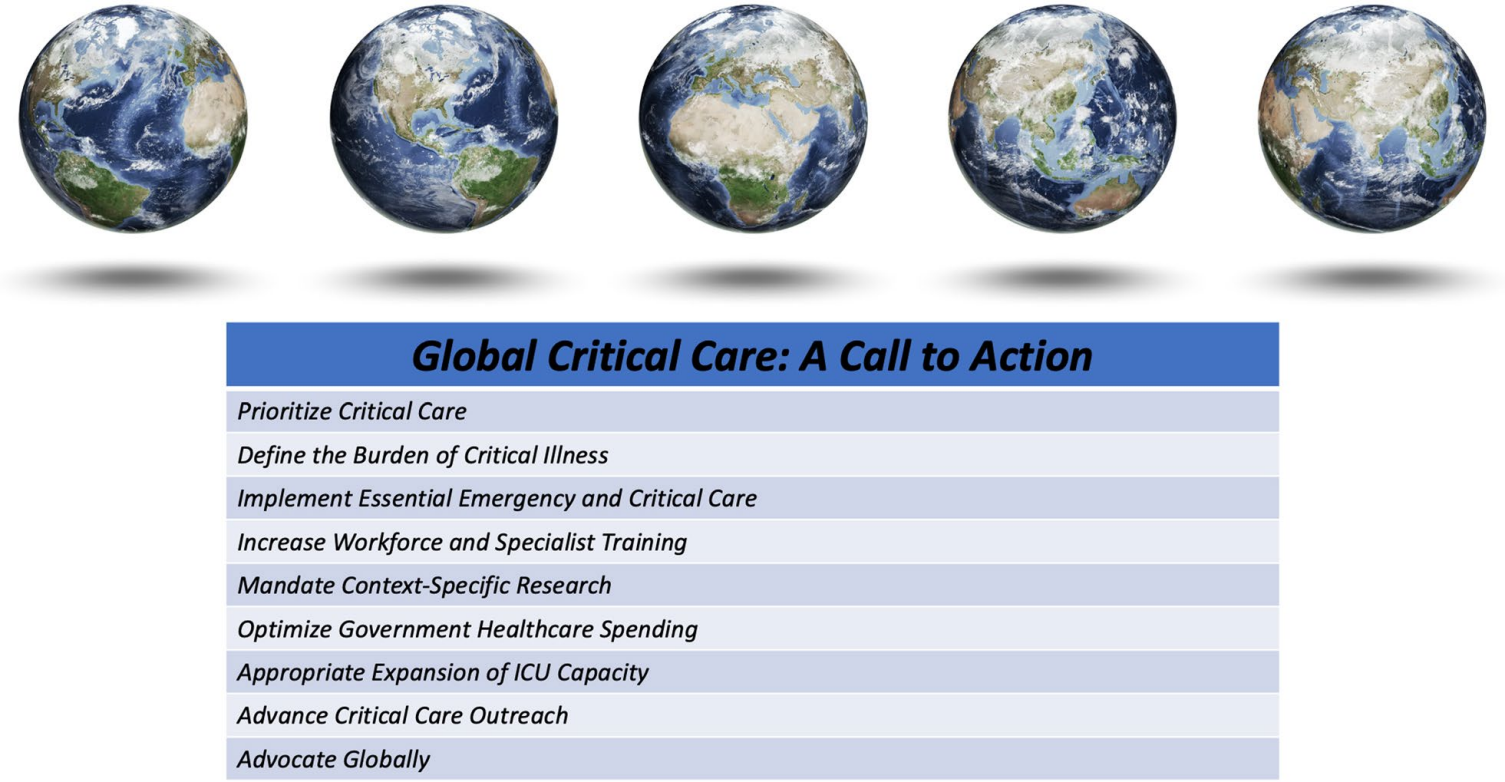
Global critical care: a call to action

This is a call to action for the global prioritization of critical care (Fig. 1). Access to critical care is needed not only to address the existing burden of critical illness, but also prepare the world for future challenges. Borrowing from the Lancet Commission on Global Surgery, critical care advocates can consider the five steps they took; creating a commission divided into core working groups, defining the current burden of disease, creating clear and concise recommendations to inform and drive policy change, develop key metrics against which to track sustainable progress, and engage in relentless advocacy until all critically ill patients receive the care they so urgently need [1].

Critical Care Medicine (CCM) has evolved drastically since its origin. In the 1850s, Florence Nightingale rearranged hospital wards placing the sickest patients closest to the nurses’ station, allowing closer observation. In the 1950s, CCM leapt forward with the polio epidemic and development of the “iron lung” used to ventilate patients with profound weakness. Almost 20 years later, in 1974, the first volume of the journal *Critical Care Medicine* was published, and the first volume of *Intensive Care Medicine* followed in 1975. CCM continues to evolve as a specialty. More than ventilators, CCM includes the care of the entire patient.

Critical illness can happen anywhere, and critical care is more than the care provided in an intensive care unit (ICU). Jean Louis Vincent framed critical illness as occurring on a continuum, stating “critical illness should be seen as just one part of the patient’s disease trajectory” [2]. He stressed that critical care requires “identifying critical illness early, before…it is life-threatening” and it requires “frequent and continuous monitoring” and a “system to call for help” with an “effective response to that call.” Care received in an ICU has an impact on the long term physical and mental outcomes of patients [2]. Unfortunately, these basic tenets are neglected in many healthcare systems, particularly in settings of resource constraint. Those with the most fragile systems carry the largest healthcare burden. Not only is mortality higher in limited-resource settings, but so too are disability and suffering [3, 4]. Any commission on critical care must be representative of those it serves.

“Critical care,” “intensive care,” “acute care,” and “emergency care” all occur on a continuum [5]. Despite multiple attempts, there is no one accepted definition of critical care. A recent concept analysis proposed a broad definition of Critical Care as “the identification, monitoring and treatment of patients with critical illness through the initial and sustained support of vital organ functions” [6]. Most agree, critical illness is severe and complex. Critical illness originates from many disease states. Cross-cutting, it affects adult, pediatric, elderly, and obstetric patients. It affects several organ systems, often simultaneously. Preventing imminent death from critical illness is taxed with complications and disability. Critical care requires a balance between meaningful survival and utilization of scare resources. It is best provided by a well-trained multidisciplinary team.

## Critical care is under-prioritized

Those who imagine intensive care units with state-of-the-art equipment, unlimited diagnostic and therapeutic options, and highly trained specialists readily available may view critical care as too costly and too resource intensive for all global settings. Often dismissed, intensive care seems too far away from current reality to be achievable. Yet, many places lack essential, low-cost, and low complexity fundamentals such as monitoring of vital signs, systems for a rapid response, and trained providers [7]. Taking care of critically ill patients is overwhelming when doctors and nurses lack adequate training, are too few, or believe nothing can be done to save the patient. While critical illness is cross-cutting, hospitals are often set up vertically with specialty-specific wards and providers.

Every system must ensure the essentials. Critical care is time-critical care, which can be delivered anywhere. Appropriate care does not always necessitate ventilators and advanced technologies. Recognition of a deteriorating patient is critical care. Early resuscitation is critical care. Basic Life Support (BLS) or cardiopulmonary resuscitation (CPR) is critical care. Further, it is not too expensive. When it originates from multiple disease states, arises in every type of patient, and occurs in all locations, critical illness prevention and treatment are cost-effective priorities.

Although not feasible or of greatest benefit in all settings, the final pathway for some critically ill patients will be an ICU. Advanced intensive care should be built upon essential systems of early recognition, vigilance, and prompt appropriate interventions. With a goal to improve patient outcomes, critical care capacity requires caring for critically ill patients along their entire continuum. Importantly, effective essential care decreases the need for advanced intensive care, potentially easing the strain on scarce ICU resources.

## The global burden of critical illness

Epidemiology for critical illness is imprecise. It stems from multiple disease states, occurs in multiple locations, and involves every type of patient. Further, the highest burden lies in the low- and middle-income countries where data are scarce. The global burden is poorly defined, and best estimates remain diagnosis-based and extrapolated. The true burden must consider critical illness and the capacity to treat critically ill patients.

Currently, the critical illness burden is characterized in its relation to ICU admissions or specific critical illness syndromes. However, a lack of ICU admissions or the lack of a certain syndrome does not mean there is a lack of critical illness. A large number of critically ill patients are located outside the ICU and a large number of places lack intensive care units. Another common measure is the amount of ICU resource utilization. However, even when ICUs are available, admission data and resource utilization patterns vary by location [8]. This is evident by use patterns in the USA versus Africa versus Latin America, for example.

The burden of critical illness is increasing. The population is aging, pandemics and epidemics continue to emerge, non-communicable disease states continue to rise, and climate change is impacting human health. Further, as care of patients and technology advances, so too will complications associated with more advanced care. One poignant example is an estimated 50% higher mortality after surgery in African patients coupled to the global priority to increase surgical volume [9].

Seven out of the 10 top causes of death are non-communicable diseases, and all seven are increasing [10]. Knowing the lack of critical care is costing lives, and some information about its burden is gleaned by examining the top 10 causes of death. Many of these diseases are responsive to early, relatively inexpensive interventions such as oxygen, intravenous fluids, antibiotics, vaccines, and insulin. Critical care is life-saving for neonatal conditions, now the 5th leading cause of death, and sadly, children are still dying of diarrheal diseases, the 8th leading cause [10].

Sepsis is a common indication for ICU admission. Measurement of serum lactic acid and the use of vasoactive medications, seen as mainstays in the management of septic shock and necessary for the international definition of septic shock, are unavailable in many resource-constrained settings. In 2017, there were 49 million cases of sepsis and septic shock. Eighty-five percent of cases and deaths occurred in low- and middle-income countries. The 11 million deaths related to sepsis in 2017 equated to 20% of total global deaths that year. Hospital and ICU mortality were 27% and 42%, respectively [11]. The ability to prevent even a fraction of 11 million deaths with time-critical interventions is incentivizing. Clearly the higher cost is sepsis, not treating it.

Acute respiratory distress syndrome (ARDS) is a leading contributor to the critical illness burden. Lower respiratory infections are the 4th leading cause of death, as the world recovers from a respiratory pandemic. Early recognition and timely intervention are key to the care of ARDS. There are problems in estimating the global burden of ARDS. First, there are multiple etiologies leading to ARDS. ARDS is often diagnosed in the ICU which many places lack. As such, the incidence is higher in the USA and Europe where ICUs are plentiful and data more robust. Another challenge is the definition of ARDS has changed over time. The incidence of ARDS may be higher in the USA and Europe, but according to the Berlin definition, ARDS cannot be diagnosed without arterial blood gas analysis and 5 cmH2O of applied PEEP. These criteria are not available in many settings. The *Kigali Modification* now makes it possible to diagnose ARDS with limited resources finding an incidence of 4% and mortality of 50% at a teaching hospital in Kigali [12]. Another group, using the Berlin definition of ARDS, observed across 5 continents, 50 countries and 459 ICUs, suggested an ICU incidence at 10.4% with ICU mortality at 35% [13]. For both sepsis and ARDS, mortality is higher and patients are younger in resource-constrained settings leading to greater social and economic impact.

Palliative care often occurs in an ICU, another proxy for the critical illness burden. In 2015, 35.5 million experienced serious health-related suffering due to life-threatening and life-limiting conditions [14]. Over 80% lived in LMICs. The *Lancet Commission on Palliative Care* predicts this burden will increase. In absolute terms, over 3 million more people will die in low-income countries with serious health-related suffering in 2060 compared with 2016, an increase of 155% [14].

Extrapolated estimates reveal over six million deaths from COVID-19, 11 million deaths from septic shock, and 2.5 million deaths due to lower respiratory tract infections [15]. There are 35.5 million palliative patients with life-threatening and life-limiting conditions. One and a half million annual deaths are attributable to diabetes [16]. There are an estimated eighteen million cardiovascular deaths each year [17]. These are snapshots of the true burden of critical illness. Just a few diagnoses demonstrate millions of annual deaths associated with critical illness conditions. In ICU, mortality reaches as high as 40% but approaches 100% for critically ill patients without access to quality care. These estimates are incomplete as they do not even account for trauma, maternal mortality, neonates, and children.

## Recommendations

Even as healthcare access improves, the quality of care received is often inadequate leaving the most vulnerable, critically ill, patients at higher risk [18]. Access and quality both must be addressed. Solutions must move past individual components of critical care and end an obsession with new technology. Targeting healthcare fundamentals such as the workforce, basic monitors, hospital processes, and essential medications stands to make a larger and more immediate impact. Essentials must be in place, or ventilators will always fail patients. Many places lack electricity and oxygen rendering ventilators useless, and while only 3–5% of COVID-19 patients needed mechanical ventilation, resource-constrained places were flooded with donated ventilators [19]. Oxygen, an essential medication critical for COVID-19 patients, was unavailable in too many places.

### Implement the essentials now

**Fig. 2:**
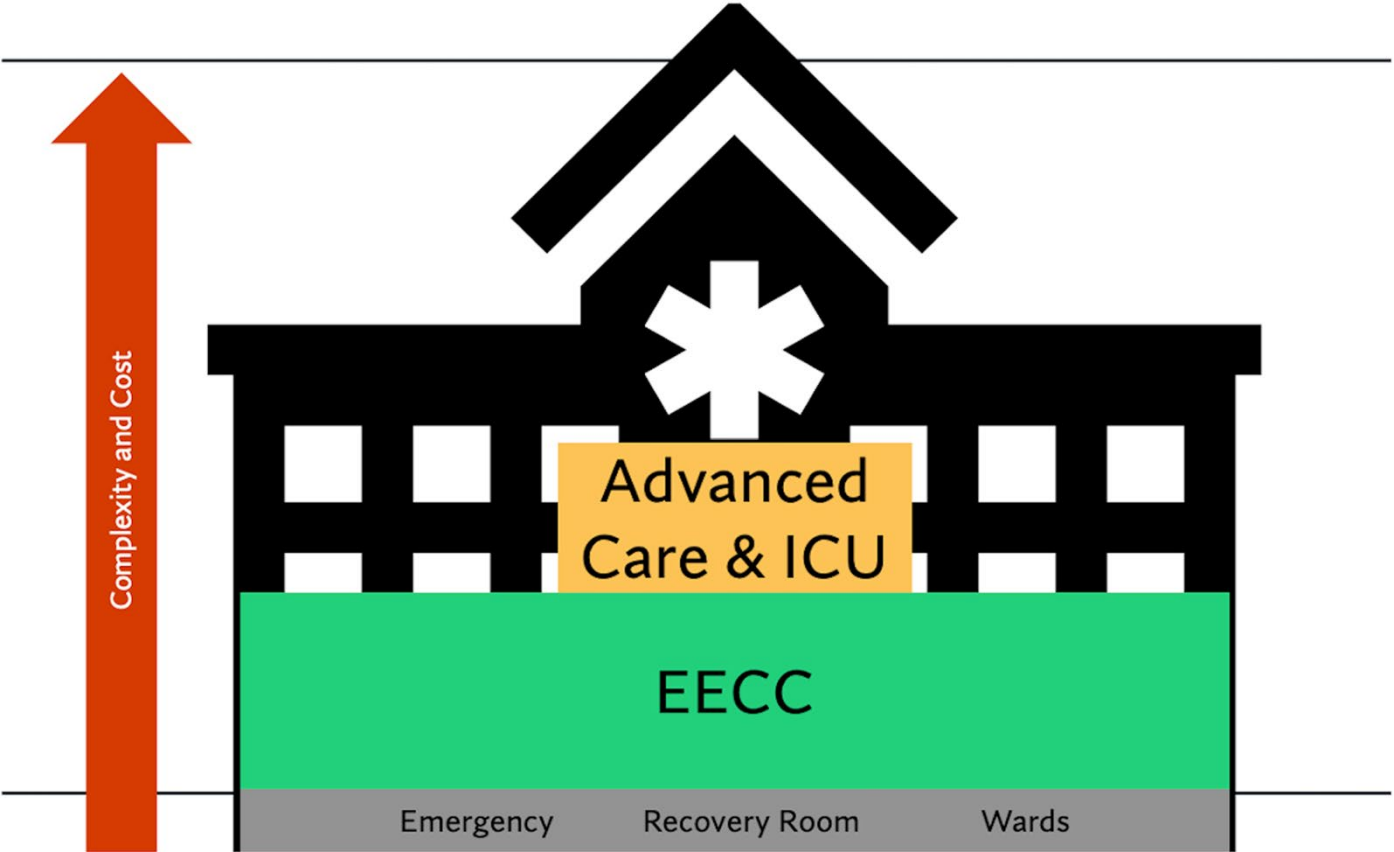
EECC is cross-cutting, improving access and quality of critical care at low cost and complexity. Adapted from Schell, C.O., Gerdin Wärnberg, M., Hvarfner, A. et al. The global need for essential emergency and critical care. Crit Care 22, 284 (2018)

It is not possible to build ICUs in every facility, but implementation of essential critical care can happen now, everywhere. The first-tier, prioritized care for critically ill patients has been specified as Essential Emergency and Critical Care (EECC) and is the care that should be available in all hospitals (Fig. 2) [7]. These essentials are mandatory for every patient everywhere to achieve UHC. Essentials consist of low-cost basic resources, providers, and processes to identify and respond to critical illness and deteriorating patients. For example, basic resources [a pulse oximeter] must be readily available to engage in essential clinical processes [mandatory vital signs checks] in order to identify critically ill patients [with hypoxemia]. Similarly, provider teams [nurses and physicians] must be ready to respond when these patients are identified [nurse call for help] and be able to intervene quickly and appropriately [oxygen therapy] [7].

Addressing both access and quality of care, EECC capacity should be in every location where patients may become critically ill. This includes wards, emergency departments, post-anesthesia recovery units, and any other patient care units. These are basic expectations of healthcare systems. These processes are not occurring reliably, a symptom of the neglect of critical care. The greater the percentage of patients identified when ill and the greater the percentage receiving an appropriate intervention, the greater the coverage of EECC. EECC coverage facilitates greater stabilization and survival for critically ill patients. EECC facilitates improved postoperative mortality, pandemic preparedness, decreased need for ICU admissions, increased workforce training, quality improvement, and increases equitable access to critical care, all at very low cost and complexity [7].

### Train the workforce

Fundamental training is needed. To improve the early recognition and first-step management of critically ill patients, early identification of critically ill patients through fundamental assessments such as vital signs checks, and physical exams must be incorporated into the curricula of all nursing and medical specialties. Additionally, well-trained critical care physician and nurse specialists are urgently needed. When empowered, specialist providers care for patients, serve as medical directors, consult on infrastructure, train additional providers, ensure quality improvement, perform research, and advance the field of critical care medicine. ICU specialists raise the quality of pre- and post-ICU care, reinforcing EECC. Intensivist-led care improves patient outcomes including mortality and length of stay [20]. Intensivist-led care improves resource utilization [21]. In some settings, intensivist-led care decreases ventilator-associated pneumonia. It pays to invest in highly trained people as costs are up to 61% higher when ICU physicians are lacking [22].

Retention is key. Healthcare systems must retain specialists who serve these invaluable functions. CCM is high stress and emotional. Burn out is real. Nurse and physician providers are leaving healthcare after the pandemic [23]. Taking care of staff in the best of times creates greater resilience to step up in the worst of times. Care of providers includes adequate time off, appropriate compensation, engagement with professional peers, continuous professional development, empowerment to improve quality, and facilitation of research.

Short learning courses are common across the globe. Post-graduate fellowship training opportunities are not. Short courses, great for continuing education, refreshers, updates, and specific topics, are inadequate for specialist training. Many short courses apply the same materials to a broad provider audience despite the disparity of resources, training, and experience. Other courses charge fees or copyright materials, even when targeting resource-constrained providers. Some short courses target single phases of care such as emergency care, triage, or trauma, with little guidance for caring for the patients’ entire clinical course or the complexity of critically ill patients. Most short course algorithms end in “transfer to an appropriate higher-level of care” that may not exist, leaving providers ill-equipped to provide effective care for the critically ill. Intensivists know that initial resuscitation or admission to the hospital or ICU is merely the beginning of a long journey taxed with avoiding complications and further deterioration. Patients reaching discharge still have significant disability as many suffer from post-intensive care syndrome with physical, cognitive, and emotional symptoms.

Ministries of health and ministries of education must invest in specialist training. Professional societies can facilitate the expeditious creation of formalized training programs, but they must be convinced critical care is a priority. Curation of curricula should preside over curricula creation, as there are many centers of excellence from which content can be adapted to local context. Academic partnerships facilitate education and training. Additionally, certification processes are necessary to ensure adequate training and core competencies that translate regionally and internationally.

### Context-specific research is required

Would you give a fluid bolus to a child in Septic Shock? Would you give a fluid bolus to an adult in Septic Shock? Can you diagnose ARDS without arterial blood gases? Can you diagnose ARDS without positive end-expiratory pressure? What is the optimal investment in critical care in low-resource settings? Answers to these questions depend on the patient population and clinical context in which they are asked. Evidence suggests that guidelines and protocols applied to one patient population may be ineffective or harmful to others. African children may fare worse with fluid boluses during sepsis, as may adults [24–26]. Context-specific research is mandatory to optimize patient outcomes and improve the quality of care delivered.

### Optimize government healthcare spending

Governments must be held accountable to invest in health and ensure those investments result in improved outcomes and appropriate utilization. In 2018, the government spending priority given to health was lowest in low-income countries, a trend that has been falling [27]. In most low-income countries, governmental health spending was between 4 and 8% of total spending, and in four low-income countries, health spending was as low as 3% [27]. Inadequate investment by governments results in several problems. Out-of-pocket spending for patients contributing to catastrophic expenditure increases as does country dependence on external aid [28]. External funding dependence relinquishes national autonomy allowing health agendas overly influenced by funders. Metrics outlined by funders do not always equate to improved patient outcomes. An equally frustrating problem occurs when spending is ineffective. The USA spends nearly twice as much as other comparable countries but has lower life expectancy, higher rates of hospitalizations, higher suicide rates, higher chronic disease burden, worse maternal outcomes, and higher infant mortality [29]. Resource-rich countries have an ethical obligation as well as practical, political, and economic reasons for taking a global perspective on critical care.

### Appropriate expansion of ICU beds

When advocating for critical care, we must be mindful of resource constraints and conflicting needs. Critical care must fit into the overall health system and must include ethical principles such as equity and justice, aiming for overall improved population health [30]. To capture the entire critical care continuum, more ICU beds are needed in some settings. Whereas the USA, Italy, and Tajikistan reported greater than 25 ICU beds per 100,000, many African countries reported a capacity of less than 1 ICU bed per 100,000 population [31]. A global mean was estimated at 8.73 beds per 100,000 population [31]. More resources do not always imply better outcomes. The USA has more ICU beds than most but was less successful in managing the pandemic, largely due to the failure of public health measures. Each setting must consider if expansion of ICU capacity is appropriate and evaluate resources for the greatest impact on the population served.

### Critical care outreach

Critically ill patients exist everywhere regardless of resource availability. Critical care outreach is needed to identify and care for as many patients as possible across this continuum. The idea of ICU outreach inside the institution is now well established. Intrahospital outreach includes the hospital wards and units, in the form of effective EECC coverage and rapid response systems. In-hospital outreach also includes consultation for critically ill patients from the perioperative period and in emergency departments.

The idea of ICU teams in central hospitals supporting smaller hospitals through “interhospital outreach” is less discussed. Critical care outreach outside the hospital can be delivered by emergency medical services such as ambulance services and patient transport services. Paramedics have been an essential part of emergency medicine teams in well-resourced countries. In Africa, they have tended to be more “scoop and run” than “stop and treat.” More critical care could be delivered out of hospital if ancillary medics could be trained to “stop, treat and deliver.” Interhospital outreach provided from tertiary healthcare centers to primary and secondary healthcare centers can educate and support the identification and initial management of critically ill patients. Telemedicine platforms are useful. The COVID-19 pandemic showed how such systems can be developed and consolidated even locally using existing platforms. Providers, regardless of practice location, should have access to Basic Life Support education and training. Bystander CPR can be taught to community members as first responders [32].

### Advocacy at all levels

Critically ill patients deserve global prioritization. Global healthcare organizations must call for critical care to be prioritized by all governments to achieve universal health coverage. Local healthcare systems must measure their burden of critical illness locally and nationally in order to inform policy decisions and research agendas. Medical providers must become advocates influencing government policy and implementation. At the institutional level, critical care teams must be formed to develop and run teaching and training projects inclusive of other disciplines. National critical care professional meetings should be multidisciplinary and representative of specialized care teams. Professional associations, including medical, nursing, and allied health, must collaboratively advocate for greater recognition of critical care medicine as a cross-cutting discipline whose underlying truth is early recognition of serious illness and early intervention whether in or out of hospitals.

## Conclusions

There is a large and growing global burden of critical illness. Critical care occurs along a continuum both inside and outside the ICU. Critical care is not an add-on, but an integral component of UHC. Countless diagnoses lead to critical illness. All patient types can require critical care. Essential Emergency and Critical Care should be made available in all primary, secondary, and tertiary centers and across all wards and units. Resource-appropriate critical care must be sufficiently available for the population served, including EECC for all and intensive care in appropriate settings. Context-specific research is needed for high-quality critical care provision. Governments must invest in critical care to prevent loss of life and ensure economic prosperity. Critical care must be prioritized by professional societies, national and regional societies, and healthcare institutions to inform and collaborate with all stakeholders. Advocacy must influence the *World Health Organization*, ministries of health, and funding agencies. Global partners have an ethical obligation as well as economic, political, and practical incentives to invest in critical care for all patients across the globe.

## Data Availability

Not applicable.

## Abbreviations

EECC: Essential Emergency and Critical Care
UHC: Universal health coverage
CCM: Critical care medicine
BLS: Basic Life Support
CPR: Cardiopulmonary resuscitation
ICU: Intensive care unit
ARDS: Acute respiratory distress syndrome
PEEP: Positive end-expiratory pressure
LMIC: Low- and middle-income countries
